# Molecular and physiological adaptations to low-salinity stress in penaeid shrimp: a focus on *Litopenaeus vannamei* and *Penaeus monodon*

**DOI:** 10.1007/s44154-026-00295-4

**Published:** 2026-04-27

**Authors:** Sheng Huang, Falin Zhou, Shigui Jiang, Erchao Li, Yundong Li

**Affiliations:** 1 Key Laboratory of South China Sea Fishery Resources Exploitation and Utilization, Ministry of Agriculture and Rural Affairs/South China Sea Fisheries Research Institute, Chinese Academy of Fishery Sciences, Guangzhou, 510300 China; 2 Key Laboratory of Efficient Utilization and Processing of Marine Fishery Resources of Hainan Province, Sanya Tropical Fisheries Research Institute, Sanya, 572018 China; 3 School of Life Sciences, East China Normal University, Shanghai, 200241 China; 4 Shenzhen Base of South China Sea Fisheries Research Institute, Chinese Academy of Fishery Sciences, Shenzhen, 518108 China; 5 Hainan Seed Industry Laboratory, Sanya, 572024 Hainan China

**Keywords:** Penaeid shrimp, Salinity stress, Molecular mechanism, Aquaculture, Signal transfer

## Abstract

Penaeid shrimp are economically important in global aquaculture. Rising demand has expanded shrimp farming and supported coastal economies. Low-salinity (hyposalinity) stress is a major constraint that impairs physiology and reduces growth, survival, and productivity. This review explores the adaptive mechanisms employed by penaeid shrimp in response to salinity stress and the consequent challenges faced by the aquaculture sector. Penaeid shrimp exhibit adaptive responses to salinity stress through a variety of physiological and molecular mechanisms, including ion regulation, osmoregulation, antioxidant defense, and the activation of signaling pathways. While these mechanisms are critical, they necessitate further in-depth investigation. This review synthesizes recent advancements in the understanding of penaeid shrimp adaptation to salinity stress, encompassing salinity signal perception, the activation of calcium and phospholipid signaling, signal transduction, and the regulation of gene expression. Additionally, it provides a comprehensive overview of the physiological and molecular responses of penaeid shrimp to salinity stress. The paper also discusses the potential implications of these findings for the aquaculture industry, particularly for improving climate-change resilience under increasingly frequent and unpredictable salinity fluctuations.

## Introduction

Penaeid shrimp, including species such as *Litopenaeus vannamei*, *Penaeus monodon*, and *Penaeus japonicus*, occupy crucial positions in the global aquaculture industry. Their economic value and ecological roles are undeniable, contributing significantly to both local economies and food security worldwide. As a highly adaptive group, penaeid shrimp can survive and reproduce across a wide range of salinities, showing broad euryhaline adaptability (Gao et al. 2016).

Despite their adaptability, salinity stress remains one of the significant environmental challenges affecting their health and productivity. With the ongoing intensification of global climate change and rising sea levels, the variability in salinity in both coastal and inland aquaculture areas has become more severe and unpredictable. This situation presents unprecedented challenges to the aquaculture industry (Li et al. 2015). Furthermore, anthropogenic activities such as excessive water extraction, desalination initiatives, and alterations in coastal land use have compounded these challenges, leading to abrupt changes in local water salinity and further destabilizing aquaculture environments (Rahi et al. 2021). Such salinity fluctuations can directly disrupt ion balance and osmoregulatory function, ultimately constraining growth, survival, and health in cultured shrimp. Linking molecular pathways (signal perception/transduction and downstream effectors) to these performance outcomes is therefore essential for building climate-resilient shrimp production systems as climate change intensifies salinity variability.

**Fig. 1:**
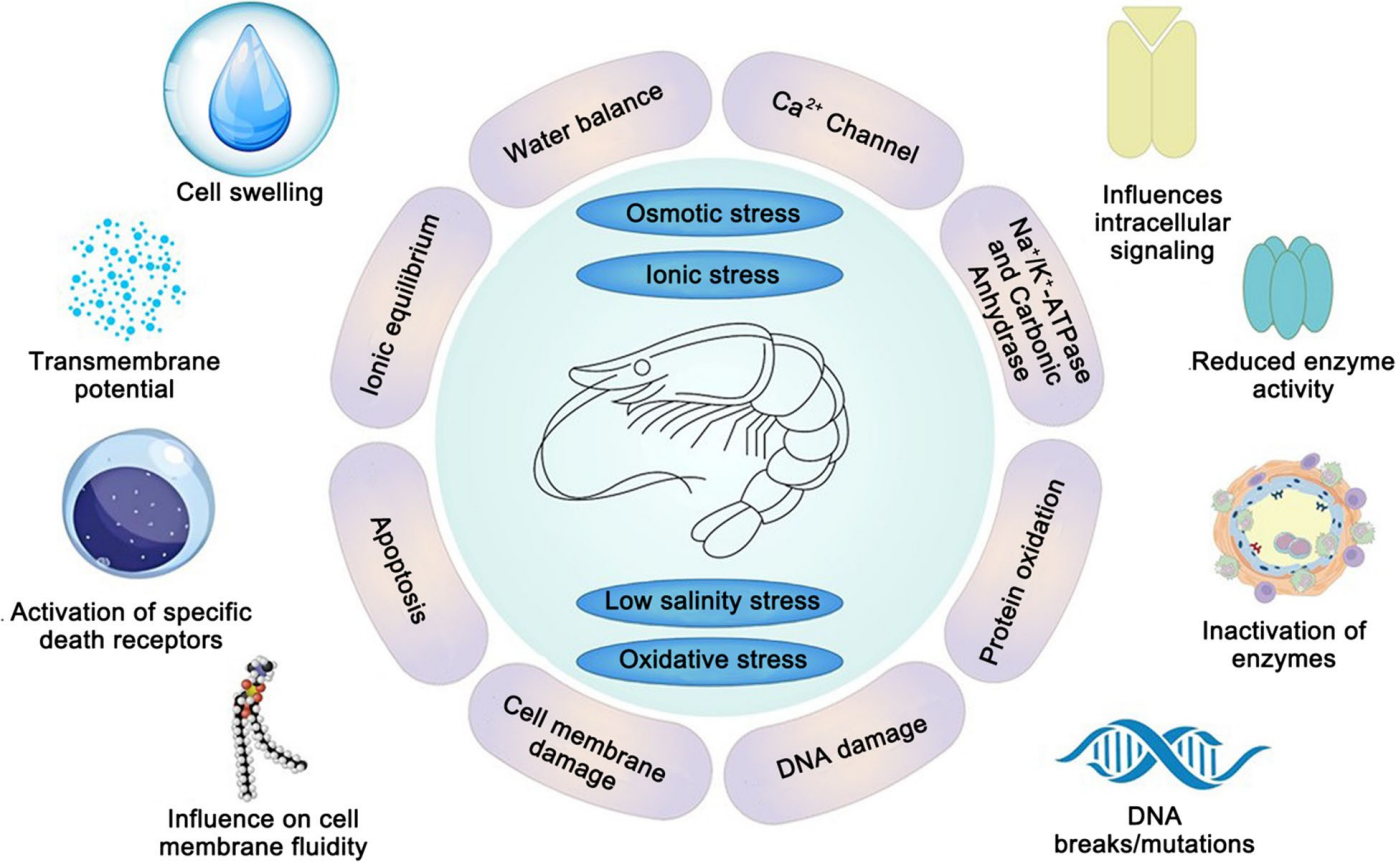
Physiological stresses in penaeid shrimp under low-salinity stress. This figure depicts the physiological stresses faced by penaeid shrimp under low salinity. Osmotic stress causes cell swelling and disrupts the water balance, whereas ionic stress impairs ion channels and enzymes critical for ionic homeostasis. Oxidative stress leads to protein oxidation, enzyme inactivation, and DNA damage, compromising cellular integrity. These stresses activate apoptosis, alter membrane fluidity, and trigger death receptor pathways, ultimately affecting shrimp health and survival

To address these challenges, penaeid shrimp activate a series of biological processes, including the regulation of ion channels and pumps, the synthesis of osmoprotectants, the activation of antioxidant defense mechanisms, and the modulation of various signaling pathways (Chen et al. 2019). At the cellular level, low salinity typically imposes combined osmotic, ionic, and oxidative challenges that can damage proteins and DNA, disturb intracellular signaling, and promote apoptosis, thereby compromising shrimp survival (Fig. 1). However, several key molecular aspects remain poorly understood. Specific gaps include the identification of upstream salinity sensors, the role of transcriptional regulators in modulating gene expression during stress, and how these processes vary across different species and genotypes (Li et al. 2015). Furthermore, the molecular pathways that integrate salinity signals into coordinated cellular responses, including ion regulation, antioxidant defense, and immune function, are still unclear. These unresolved questions hinder a mechanistic interpretation of salinity acclimation.

This review provides a focused synthesis of recent advances in the molecular mechanisms underlying salinity tolerance in penaeid shrimp, with particular emphasis on *L. vannamei*. Where available, evidence from other penaeid species (e.g., *P. monodon* and *Marsupenaeus japonicus*) is incorporated for comparison to highlight conserved versus species-specific features of salinity acclimation. Given the current literature distribution, this review focuses primarily on low-salinity (hyposalinity) stress; high-salinity stress and rapid salinity shifts are discussed only where shrimp-specific evidence is available. It summarizes salinity signal perception (including calcium and phospholipid signaling), downstream signal transduction, and gene expression involved in adaptation. Furthermore, this review integrates physiological and molecular responses to salinity stress, including physiological regulation, the function of autophagy proteins, endoplasmic reticulum (ER) stress responses, and interactions between immune-related proteins and salinity stress. Collectively, the evidence synthesized in this review clarifies molecular strategies employed by penaeid shrimp to cope with salinity stress, thereby providing scientific insights and potential applications for penaeid shrimp aquaculture. Finally, this review outlines future research directions and challenges, including resolving mechanistic details, improving aquaculture technologies, understanding intra-/inter-species variation, and advancing breeding approaches for salinity tolerance.

## Salinity signal perception

### Activation of GPCRs under salinity stress

G protein-coupled receptors (GPCRs) are widely represented in penaeid shrimp genomes and transcriptomes (Sun et al. 2020). In the *L. vannamei* reference genome, 457 GPCR genes were annotated (Zhang et al. 2019), and transcriptome-based curation in *P. monodon* identified three major GPCR classes—rhodopsin-like (Class A), secretin-like (Class B), and metabotropic glutamate-like (Class C)—with 223, 100, and 27 members, respectively (Viet Nguyen et al. 2020).

Upon low-salinity exposure, environmental dilution alters extracellular ion composition and osmolyte conditions (Mo et al. 2024). In penaeid shrimp, direct evidence that GPCRs act as primary salinity sensors remains limited; instead, GPCR pathways are more plausibly engaged indirectly via ligand-dependent mechanisms, whereby salinity shifts alter neuroendocrine/paracrine ligand availability or receptor–ligand interactions (Zhao et al. 2016). After ligand-driven activation, GPCRs signal through G proteins to modulate downstream pathways and effector enzymes such as adenylate cyclase (cAMP production) and phosphoinositide 3-kinase (Kim et al. 2022b). This dissociation process subsequently influences multiple downstream signaling molecules and transcription factors, including cyclic adenosine monophosphate (cAMP), protein kinase A (PKA), and mitogen-activated protein kinase (MAPK), which regulate the expression of genes related to the salinity stress response (Kim et al. 2022a). Consistently, transcriptomic profiling of *P. monodon* gills under chronic low-salinity stress showed that differentially expressed genes significantly mapped to multiple signal-transduction pathways, including PI3K–Akt, MAPK, and calcium signaling pathways (Li et al. 2023), supporting the involvement of GPCR-linked cascades in gill-centered osmoregulatory acclimation.

Although GPCR-linked signaling is implicated in salinity acclimation, shrimp-specific evidence pinpointing particular GPCR genes as direct upstream osmosensors remains limited. Current omics studies mainly support pathway-level engagement of GPCR-associated cascades (e.g., cAMP/PKA, PI3K–Akt, MAPK, and calcium signaling) during low-salinity challenge rather than demonstrating direct ionic sensing by specific receptors. Therefore, identifying candidate GPCR subfamilies, validating ligand–receptor pairs, and establishing causal roles using functional assays (e.g., RNA interference (RNAi)/CRISPR-based perturbation coupled with physiological readouts) remain important future directions.

### Activation of calcium signaling under salinity stress

**Fig. 2:**
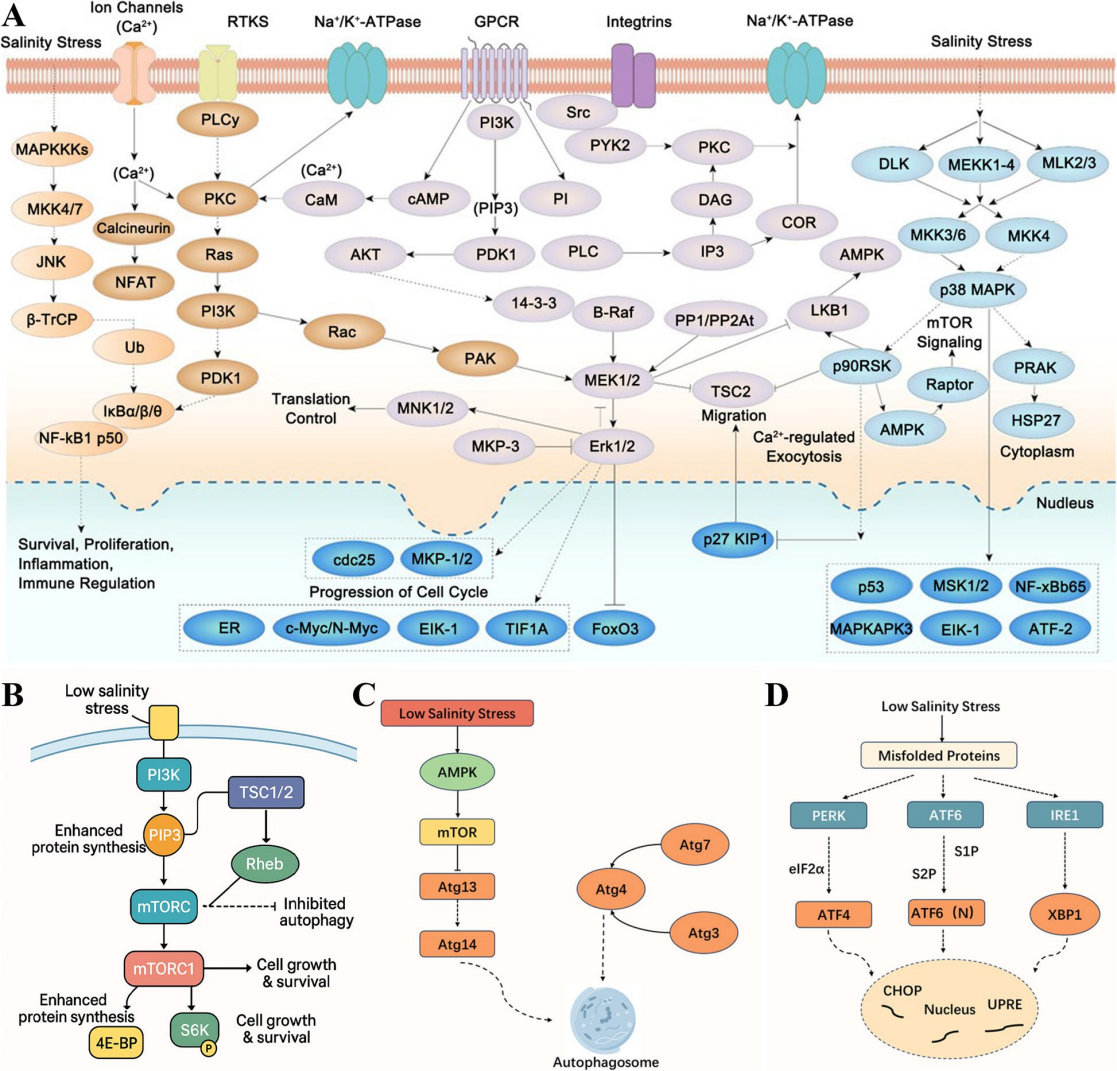
Molecular pathways affected by low-salinity stress in Penaeid Shrimp. **A** Early membrane-to-nucleus signal map (overview). Ionic and mechanical cues at the cell surface engage ion channels, RTKs, Na⁺/K⁺-ATPase, GPCRs and integrins, funneling signals through CaMK/CaN, PKC, PI3K-Akt, Ras–MAPK (ERK, JNK, p38) and AMPK branches to coordinate survival, inflammation, translation control and cell cycle regulation. **B** PI3K–Akt–mTOR growth axis. Low salinity activates PI3K, generating PIP₃ and recruiting mTORC via the Rheb GTPase. Active mTORC1 phosphorylates 4E-BP, increasing its inhibition of eIF4E and S6K to increase cap-dependent translation and promote cell growth and survival, whereas the TSC1/2 complex provides negative feedback that limits autophagy (dashed inhibitory line). **C** AMPK-driven autophagy cascade. Energetic stress associated with hypo-osmotic challenge stimulates AMPK, which suppresses mTOR activity and releases the Atg13–Atg14 complex. Subsequent recruitment of Atg4, Atg7 and Atg3 catalyzes Atg8 lipidation and elongation of the phagophore membrane, culminating in autophagosome formation for damaged-organelle turnover. **D** Endoplasmic–reticulum unfolded–protein response (UPR). The accumulation of misfolded proteins activates three sensor arms: PERK (phosphorylates eIF2α to favor ATF4 translation), ATF6 (S1P/S2P-mediated nuclear cleavage to yield ATF6 [N]), and IRE1 (splices XBP1 mRNA). The transcription factors ATF4, ATF6 [N] and XBP1 converge on CHOP and UPRE elements to restore folding capacity or trigger apoptosis if stress persists

Under low-salinity stress in *L. vannamei*, Ca^2^⁺ signaling represents a coordinated signaling response that supports homeostasis and acclimation (Li et al. 2023). As salinity decreases, leading to reduced extracellular osmotic pressure, osmotic and ionic perturbations may engage multiple Ca^2^⁺ entry/handling routes (Fig. 2A), including Ca^2^⁺-permeable membrane channels/transporters and mobilization of Ca^2^⁺ from intracellular stores (Huang et al. 2019a). Consistent with Ca^2^⁺ involvement in shrimp, acute low-salt stress in *Penaeus monodon* significantly enriched gene sets related to ‘calcium ion transmembrane transport’, ‘calmodulin binding’, and ‘regulation of cytosolic calcium ion concentration’, and was accompanied by increased activity/expression of Ca^2^⁺/Mg^2^⁺-ATPase (Fig. 2A) (Li et al. 2025). Once these channels detect external environmental changes, they open, allowing the influx of calcium ions from the external environment or the release of calcium ions from internal stores, such as the endoplasmic reticulum, into the cytoplasm (Shekhar et al. 2014). This leads to a transient increase in the intracellular calcium concentration, initiating a cascade of downstream responses (Pan et al. 2019). In *L. vannamei*, multi-omics analysis of a low-salinity selected population further reported up-regulation of genes associated with calcium-activated chloride channels under low-salinity conditions, supporting a Ca^2^⁺-dependent effector link to ion-transport remodeling (Ye et al. 2025).

A transient increase in cytosolic Ca^2^⁺ activates a broad set of Ca^2^⁺-sensitive kinases and phosphatases that fine-tune ion pumps and channels, thereby helping the cell reestablish ionic and osmotic homeostasis. (Proietti Onori and van Woerden 2021). These kinases and proteins further modulate the activity of ion pumps and channels through phosphorylation, regulating the cellular ion balance and osmotic pressure to adapt to reduced salinity conditions (Blaustein et al. 2020). This process is crucial not only for restoring the calcium ion balance but also for maintaining the equilibrium of other key ions, such as potassium and sodium, ensuring comprehensive cellular functionality (Demaurex et al. 2009). Notably, the cellular energy sensor AMPK is activated in parallel with the Ca^2^⁺ wave: hepatopancreatic AMPK-α transcripts increase ~ 28-fold just 6 h after Pacific white shrimp are transferred from Salinity 20 to Salinity 3 (Xu et al. 2016). Mechanistically, Ca^2^⁺ signals can be coupled to energy sensing through Ca^2^⁺/calmodulin-dependent pathways (e.g., Ca^2^⁺/calmodulin-dependent protein kinase kinase (CaMKK)-mediated adenosine monophosphate-activated protein kinase (AMPK) activation), thereby linking osmotic Ca^2^⁺ dynamics to metabolic reprogramming. In shrimp, low salinity elevates AMPK expression in the hepatopancreas and is accompanied by downregulation of mTOR-associated molecules, supporting engagement of an AMPK–mechanistic target of rapamycin (mTOR) during hyposalinity acclimation (Cheng et al. 2025).

### Phospholipid signaling

When *L. vannamei* is subjected to low salinity stress, the reduction in environmental salinity and the consequent change in extracellular osmotic pressure directly affect the physical state of the cell membrane, particularly its fluidity and the activity of membrane proteins (Cheng and Chen 2000). This stress induces adjustments in the composition and state of membrane phospholipids, a crucial cellular response mechanism to external salinity changes (Fig. 2A) (Li et al. 2024b). Importantly, phospholipid metabolism under low salinity should be viewed primarily as membrane remodeling that stabilizes membrane integrity and preserves the function/trafficking of osmoregulatory membrane proteins (e.g., ion channels and pumps) during cell swelling and volume recovery. During this process, phospholipid turnover can be activated through phospholipases (e.g., phospholipase C (PLC)), and PLC-mediated hydrolysis of phosphoinositides can generate second messengers such as inositol 1,4,5-trisphosphate (IP3) and diacylglycerol (DAG) (Mykles 2021).

Rather than emphasizing the generic PLC–IP3/DAG functions, shrimp-relevant phospholipid signaling can be summarized as lipid turnover that couples membrane remodeling to osmotic regulation. Specifically, changes in phosphoinositide pools and DAG/PKC-linked signaling can tune membrane–protein interactions and the activity/trafficking of ion-transport systems, thereby facilitating ion balance restoration and low-salinity acclimation (Noor et al. 2020).

Under long-term low salinity stress, penaeid shrimp also adjust the composition of cell membrane phospholipids to increase membrane fluidity, particularly by altering the ratio of saturated to unsaturated fatty acids (Huang et al. 2019b). This remodeling helps maintain membrane permeability and the functional conformation/localization of osmoregulatory transport proteins during sustained hypo-osmotic challenge. The increase in unsaturated fatty acids maintains necessary membrane fluidity and flexibility under low salinity stress, which is crucial for the functionality of membrane proteins, especially those involved in signaling, such as receptors and channels. Additionally, this adjustment in membrane fluidity helps maintain the ability of the cell to perceive external signals and the effectiveness of signal transmission (Long et al. 2017).

Together, these salinity-perception modules (GPCR-, Ca^2^⁺-, and phospholipid-associated signaling) translate extracellular osmotic/ionic perturbations into intracellular second-messenger and membrane/energy cues that converge on downstream kinase and transcriptional networks, as summarized in Section "Signal Transduction And Gene Expression Regulation".

## Signal transduction and gene expression regulation

Downstream of the salinity-perception events outlined in Section "Salinity Signal Perception", given the crosstalk and partial overlap among the MAPK (ERK, extracellular signal-regulated kinase; JNK, c-Jun N-terminal kinase/p38), PI3K–Akt (protein kinase B), and NF-κB (nuclear factor kappa B) pathways in upstream activation, downstream effectors, and physiological outcomes, the key elements of these modules are consolidated in Table 1 for side-by-side comparison of core regulatory nodes and shrimp-relevant responses under low-salinity stress. The following subsections build on this summary by highlighting the most relevant shrimp evidence and potential interactions among pathways during hyposalinity acclimation. Across these pathways, the major functional endpoints during hyposalinity acclimation include gill-centered osmoregulation (ion transport and volume regulation), immune priming/defense programs, and antioxidant/reactive oxygen species (ROS)-control responses, which are expanded in Sections "Physiological And Molecular Responses Of Penaeid Shrimp To Low-Salinity Stress"–"Immune and antioxidant responses to low-salinity stress".

**Table 1:** Key signaling pathways involved in hyposalinity response in penaeid shrimp

Pathway	Key regulators/nodes	Representative downstream outputs	Shrimp-relevant physiological outcomes
MAPK family (ERK/JNK/p38)	MAPKKK → MAPKK → MAPK; ERK1/2; JNK; p38; MKK4/7; MKK3/6; Ras/MEK	AP-1 (c-Jun), ATF-2/Max; stress-response gene programs; crosstalk to NF-κB	Growth/proliferation (ERK); stress adaptation/antioxidant responses; apoptosis under severe stress (JNK/p38) (He et al. 2005; Jiang et al. 2020; Wang et al. 2022b); inflammatory signaling modulation (Fan et al. 2021; Huang et al. 2017; Aweya et al. 2022)
PI3K–Akt (± mTOR/FOXO)	PI3K; PIP3; Akt; PTEN; mTOR; FOXO	Survival/metabolism programs; anti-apoptotic regulation	Cell survival and homeostasis; metabolic reprogramming; growth/translation control via mTOR; apoptosis restraint via FOXO/Bad (Su et al. 2014; Hsieh et al. 2015; Hu et al. 2020)
NF-κB	IκBα; IKK; NF-κB nuclear translocation; MAPK/PI3K inputs	Immune/inflammatory gene expression; anti-apoptotic genes	Immune activation and stress resistance; anti-apoptotic/survival gene regulation under low salinity (Qiu et al. 2017; Yang et al. 2020; Yin et al. 2022)

### MAPK signaling pathway under salinity stress in penaeid shrimp

The MAPK pathway is a central stress-response pathway and contributes to salinity acclimation in *L. vannamei* (Fan et al. 2021). This pathway encompasses a series of kinase cascades that sequentially activate MAPKs, thereby regulating the expression of specific genes that influence cell growth, differentiation, and survival under environmental stress (Hu et al. 2015). As shown in Fig. 2A, the MAPK pathway is divided into three major branches, namely, the ERK, JNK, and p38 MAPK branches, each of which corresponds to different stimuli and functions (Huang et al. 2017).

Under environmental stresses such as salinity stress, temperature changes, and mechanical damage, the MAPK pathway is activated, initiating kinase cascades where MAPKKK activates MAPKK, which in turn activates MAPK (Yan et al. 2017). This process is primarily facilitated through phosphorylation, ultimately conveying signals to the nucleus to activate specific transcription factors (such as NF-κB), influencing the biological responses of cells to external stresses (Li et al. 2023).

Among these branches, the ERK pathway primarily responds to growth factors and mitogenic signals and is activated through Ras proteins to activate MEK1/2, which then activates ERK1/2, promoting cell division and proliferation. The JNK pathway, which is activated by stress and inflammatory (Chen et al. 2018b) factors, hinges on the phosphorylation of MKK4/7, which activates JNK, leading to the phosphorylation of c-Jun, which amplifies stress-responsive genes that regulate genes involved in stress responses (He et al. 2013). The p38 MAPK pathway responds to environmental stress and is activated through MKK3/6 to regulate p38 MAPK, which influences transcription factors such as ATF-2 and Max, affecting genes related to inflammation, cell differentiation, and apoptosis (Feng et al. 2022).

These pathways are indispensable for cell growth and differentiation, stress responses, and inflammatory reactions. The ERK pathway supports cell growth and differentiation, whereas the JNK and p38 MAPK pathways induce apoptosis under severe environmental stress, eliminating damaged cells (Aweya et al. 2022). The p38 MAPK pathway also participates in the inflammatory process and immune responses by regulating the expression of inflammatory factors (He et al. 2018). Within the MAPK family, the JNK branch is frequently associated with stress-responsive transcription (e.g., AP-1/c-Jun–related outputs) and apoptosis/antioxidant programs in shrimp under hyposalinity, and is therefore discussed here as a MAPK sub-branch (Table 1). Functionally, MAPK outputs provide a link from salinity-triggered stress signaling to adaptive phenotypes, including regulation of osmoregulatory transport processes in gill epithelia (e.g., ion uptake and acid–base adjustment), coordination of immune/inflammatory gene expression, and modulation of antioxidant/stress-response programs under hyposalinity.

### PI3K/Akt signaling pathway and its gene regulatory mechanisms under low-salinity stress

Under low-salinity stress, the phosphoinositide 3-kinase/protein kinase B (PI3K/Akt) pathway plays a crucial role as one of the core signaling pathways regulating cell survival, proliferation, and antistress responses (Su et al. 2014). This pathway is typically activated by extracellular signals, such as growth factors or other environmental stimuli, including low salinity stress, initiating the activation of PI3K (Fig. 2A) (Hsieh et al. 2015).

Once PI3K is activated, it catalyzes the production of phosphatidylinositol (3,4,5)-trisphosphate (PIP3), which serves as a key messenger to activate Akt (protein kinase B) (Li et al. 2023). The activation of Akt triggers a series of downstream effects. Once Akt is engaged, it phosphorylates the mechanistic target of rapamycin (mTOR), promoting anabolic metabolism and cell growth. These effects not only promote cell survival and proliferation but also prevent programmed cell death by inhibiting proteins in apoptotic pathways, such as the Bcl-2-associated death promoter (Bad) (Li et al. 2013).

In addition to the genes mentioned above, the PI3K/Akt pathway also regulates the expression and activity of several other key genes and proteins, such as the FOXO family of transcription factors (Chaichanit et al. 2018). When unphosphorylated by Akt, FOXO proteins enter the nucleus and promote the expression of cell cycle inhibitors and apoptosis-related genes. The activation of Akt phosphorylates members of the FOXO family, resulting in their exclusion from the nucleus and suppression of their transcriptional activity, thereby promoting cell survival over apoptosis (Li et al. 2024a).

Under low-salinity stress, the activation and regulatory functions of the PI3K/Akt pathway are essential for maintaining cellular homeostasis and protecting cells from stress-induced damage. Additionally, the internal negative feedback regulatory mechanisms of the pathway, such as the dephosphorylation of PIP3 by phosphatase and tensin homolog (PTEN), ensure a dynamic balance and appropriate response of the signaling pathway, avoiding pathological changes due to overactivation (Hu et al. 2020). At the organismal level, this survival/energy-allocation role is consistent with the high ATP demand of gill-centered osmoregulation under low salinity, where sustaining ion-transport activity and tissue integrity is essential for restoring hemolymph ion balance during acclimation (see Section "Physiological And Molecular Responses Of Penaeid Shrimp To Low-Salinity Stress").

### NF-κB signaling pathway and its gene regulatory mechanisms under low-salinity stress

In aquatic organisms, particularly in species such as *L. vannamei*, which faces low salinity stress, the activation and regulation of the nuclear factor kappa-light-chain-enhancer of activated B cells (NF-κB) pathway play crucial roles (Qiu et al. 2017). This pathway is a key regulatory pathway for cellular responses to various stresses, including changes in salinity, and it responds to environmental pressures through molecular mechanisms to maintain cellular survival and development (Fig. 2A) (Wang et al. 2013).

As outlined in Table 1, low-salinity stress can engage NF-κB signaling via IκB regulation and nuclear translocation, thereby promoting immune/stress-response and anti-apoptotic gene programs in penaeid shrimp (Yang et al. 2020). Under low-salinity stress, the regulatory role of the NF-κB pathway is particularly significant, as it not only enhances cell defense mechanisms by promoting the expression of inflammation and immune response genes but also helps cells adapt to low-salinity environments by regulating the expression of antiapoptotic and cell survival genes, thereby mitigating the physiological impacts of stress (Yin et al. 2022).

Collectively, these signaling modules translate salinity perception into coordinated functional adaptation outcomes in penaeid shrimp: (i) activation of gill-centered osmoregulatory machinery to restore ion and acid–base balance, (ii) survival and energy reallocation to sustain the cost of ion transport and limit cellular damage, and (iii) coupling of innate immunity with antioxidant defense to counter ROS accumulation and reduce disease susceptibility under hyposalinity. These pathway-to-phenotype links are detailed in Sections "Physiological And Molecular Responses Of Penaeid Shrimp To Low-Salinity Stress" (physiological regulation and key transporters) and 5 (immune and antioxidant responses).

## Physiological and molecular responses of penaeid shrimp to low-salinity stress

Building on the perception layer (Section "Salinity Signal Perception") and the signal-transduction layer (Section "Signal Transduction And Gene Expression Regulation"), penaeid shrimp deploy organ- and tissue-level effector mechanisms to re-establish osmotic and ionic balance under salinity stress.

### Physiological regulatory mechanisms

**Fig. 3:**
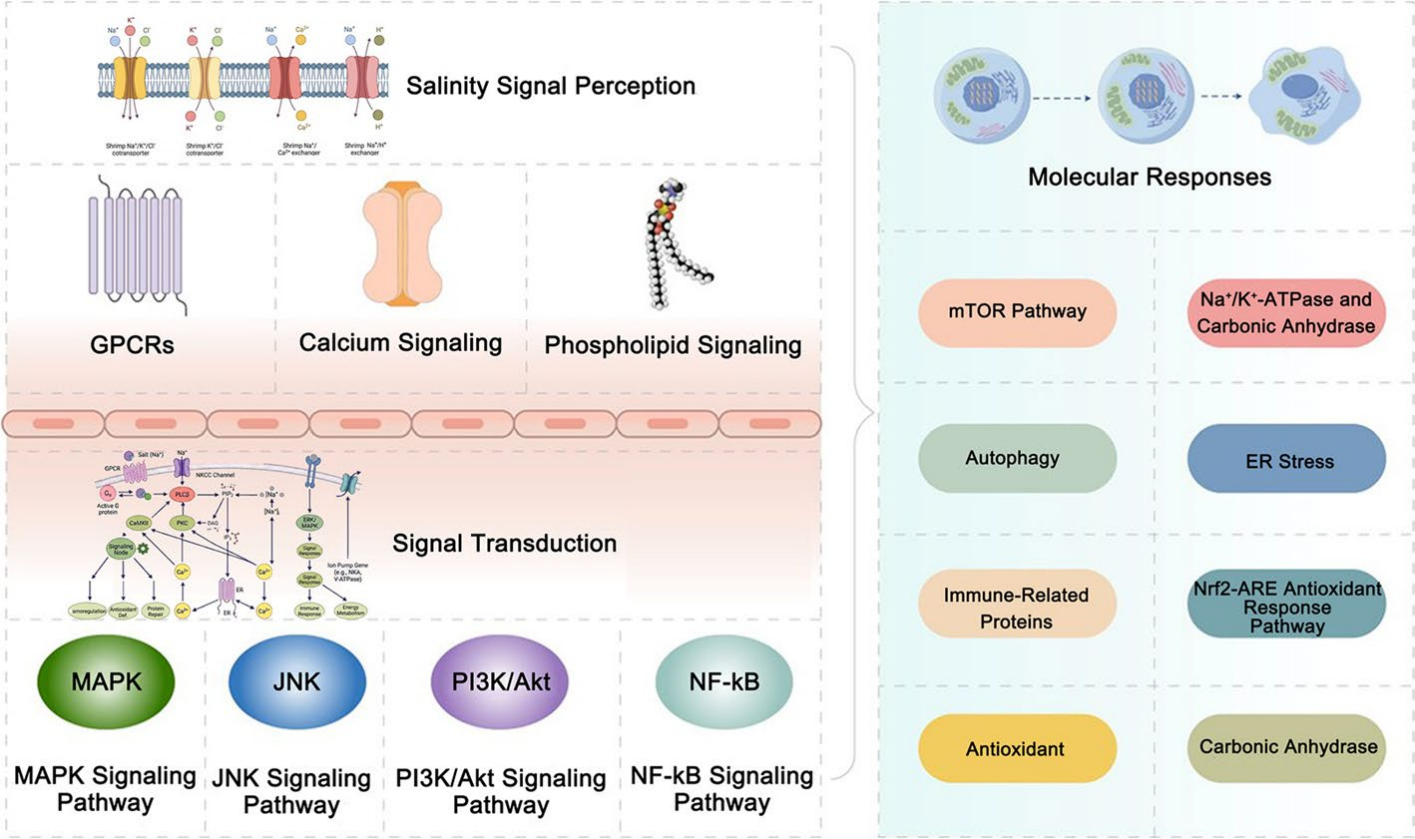
Overview of salinity stress response mechanisms in penaeid shrimp. This figure illustrates how penaeid shrimp respond to low salinity stress. Key signaling pathways, including G-protein coupled receptors (GPCRs), calcium channels, and phospholipid signaling, detect salinity changes and activate transduction pathways such as the MAPK, JNK, PI3K/Akt, and NF-κB pathways. These pathways trigger molecular adaptations, such as mTOR, Na⁺/K⁺-ATPase, and carbonic anhydrase, for ion balance, autophagy and ER stress responses for cellular integrity, and antioxidant defenses (e.g., the Nrf2-ARE pathway) to combat oxidative stress, helping shrimp maintain homeostasis under low-salinity conditions

Penaeid shrimp use gills, antennal glands, and maxillary glands for osmoregulation. At the molecular level, these tissue outputs are executed by ion-transport epithelia and excretory structures whose transporter expression/activity is tuned by the salinity-perception and signal-transduction modules summarized in Sections "Salinity Signal Perception"–"Signal Transduction And Gene Expression Regulation" (GPCR/Ca^2^⁺/phospholipid signaling and MAPK/PI3K–Akt/NF-κB networks). In this process, the gills play a central role in which the ion-transporting epithelial cells dynamically adjust the absorption or excretion of ions on the basis of changes in environmental salinity. These gill ionocytes implement osmoregulation through coordinated regulation of core transport systems (e.g., Na⁺/K⁺-ATPase and carbonic anhydrase; see Section "Na^+^/K^+^-ATPase and carbonic anhydrase"), together with other ion-uptake/acid–base regulators that collectively restore hemolymph ion balance under hyposalinity. In low-salinity environments, *P. monodon* enhances ion uptake through its gills to increase internal ion concentrations and thereby increase osmotic pressure within the body, effectively preventing excessive water absorption and subsequent cellular rupture. Conversely, in high-salinity environments, *P. monodon* enhances ion excretion to decrease internal osmotic pressure, adapting to increased salinity levels. The involvement of the antennal and maxillary glands further enhances this regulatory mechanism by precisely controlling the excretion of water and ions, aiding in maintaining osmotic balance within the body. This adaptive adjustment of specific organ structures and functions demonstrates the high adaptability of *M. jelskii* to changes in salinity (Fig. 3) (Mantovani and McNamara 2021).

The antennal glands play crucial auxiliary roles in *L. vannamei* osmoregulation. These glands primarily handle the excretion of excess water in low-salinity environments and the excretion of excess ions in high-salinity conditions. Mechanistically, antennal-gland outputs are coordinated with gill ion transport through shared second-messenger and kinase networks (e.g., cAMP/PKA-, Ca^2^⁺-linked signaling and MAPK/PI3K–Akt nodes), which tune transporter activity and epithelial transport capacity during acclimation. The function of the antennal glands is facilitated by a series of signaling pathways and transcriptional regulatory networks. For example, under low-salinity conditions, antennal glands increase the expression and activity of specific ion channels and pumps, such as Na^+^/K^+^-ATPase, to promote ion reabsorption and water excretion, thus preventing cellular rupture due to excessive water absorption. In this process, intracellular signaling molecules such as cAMP may play a regulatory role, enhancing the functionality of ion pumps and channels through the activation of specific signaling pathways (Wang et al. 2012).

The maxillary glands also contribute to osmoregulation in *L. vannamei*, especially in buffering hemolymph osmotic/ionic shifts during salinity stress. Current evidence suggests that their regulation likely relies on molecular effectors similar to those in gills and antennal glands (ion pumps/channels and their control via second messengers and kinase pathways), although shrimp-specific mechanistic data remain limited. These physiological adjustments have direct implications for aquaculture, where maintaining gradual salinity transitions and minimizing osmotic shocks are crucial strategies to support the ion-regulatory capacity of shrimp and reduce mortality during environmental fluctuations. The maxillary glands participate in regulating the internal balance of water and ions by adjusting their secretory activity. In decapod crustaceans, the antennal/maxillary (green) gland functions as an excretory organ contributing to ionic and osmotic homeostasis; however, shrimp-specific mechanistic evidence for maxillary-gland effector molecules and signaling control remains limited. Accordingly, this section is restricted to a tissue-level role and avoids over-interpreting secreted ‘metabolic products’, pending direct physiological and molecular validation (Kruangkum et al. 2024).

Recent transcriptome analysis of *P. monodon* under salinity fluctuations revealed significant modulation of osmoregulatory genes. In particular, the expression of Na⁺/K⁺-ATPase subunits was markedly upregulated under low salinity stress, which is consistent with the enhanced active ion uptake observed at the physiological level (Huang et al. 2025). Similarly, carbonic anhydrase (CA) transcripts were significantly elevated, supporting the role of acid‒base balance regulation during osmotic adjustment. Furthermore, genes related to cytoskeletal remodeling, such as actin and tubulin family members, showed dynamic changes, indicating active cellular structural reorganization in response to osmotic gradients. These molecular responses correspond with the physiological observations of gill epithelial remodeling and improved ion transport efficiency depicted in Fig. 3 (Huang et al. 2025).

### Activation of the mTOR pathway

Experimental evidence shows that shifting shrimp from salinity 20 to salinity 3 elevates hepatopancreatic AMPK protein by ~ 1.6-fold within 6 h (P < 0.05), whereas muscle AMPK returns to baseline by 12 h, underscoring a rapid yet tissue-specific energy-sensing response (Xu et al. 2016). Once AMPK has stabilized the cellular energy budget, it controls transitions to downstream anabolic regulators—most notably, the mTOR pathway, which integrates nutrient cues with growth and repair programmes.

In the molecular response of *L. vannamei* to low salinity stress, the mammalian target of rapamycin (mTOR) pathway plays a central role in regulating *L. vannamei* growth, metabolism, and survival mechanisms through a series of finely tuned steps (Zhao et al. 2021b). The mTOR pathway is a highly conserved cellular growth and metabolism regulatory pathway whose activity is influenced by various environmental signals, including nutritional status, energy state, and stress conditions (Su et al. 2014). Under low-salinity stress, *L. vannamei* adapts to environmental changes by modulating the mTOR pathway to maintain physiological balance and promote survival (Fu et al. 2017).

When *L. vannamei* faces low salinity stress, initially, changes in extracellular salinity are recognized by cell surface receptors, which transmit external salinity stress signals into the cell through a series of signaling molecules. The key signaling molecules involved include phosphoinositide 3-kinase (PI3K) and protein kinase B (Akt). Upon receiving stress signals, PI3K is activated and produces PIP3 (phosphatidylinositol-3,4,5-trisphosphate), a secondary messenger that further activates Akt. The activation of Akt, achieved through its phosphorylation, is crucial for the subsequent activation of mTOR (Xiao et al. 2023).

Following Akt activation, it directly acts on mTOR 1 (mTORC1), inhibiting the activity of TSC1/2 (tuberous sclerosis 1/2) through phosphorylation, thereby relieving the inhibition of Rheb (Ras homolog enriched in brain GTPase) (Liu et al. 2023). The GTP-active state of Rheb directly activates mTORC1, promoting downstream signal transmission. Activated mTORC1 coordinates anabolic growth (e.g., protein synthesis via 4E-BP/S6K) and suppresses autophagy, thereby shaping cell growth and stress recovery. Under hyposalinity, this module is thought to help maintain tissue function and limit damage by balancing anabolic repair with catabolic recycling (Fig. 2B) (Rabanal-Ruiz et al. 2017).

In the mTOR signaling pathway, key regulatory proteins such as 4E-BP (eIF4E-binding protein) and S6K (ribosomal protein S6 kinase) are also involved (Zhao et al. 2021b). The activation of mTORC1 directly phosphorylates 4E-BP, increasing its inhibition of eIF4E and thereby promoting protein synthesis. Simultaneously, the phosphorylation of S6K by mTORC1 enhances the phosphorylation of the ribosomal protein S6, further promoting protein synthesis and cell growth (Fig. 2B) (Luo et al. 2024).

### Na^+^/K^+^-ATPase and carbonic anhydrase

In *L. vannamei* facing low salinity stress, the Na^**+**^/K^**+**^-ATPase and carbonic anhydrase (CA) pathways are key physiological regulatory systems that work together through precise molecular mechanisms to maintain the intracellular and extracellular ion balance and acid‒base equilibrium, thereby facilitating *L. vannamei* adaptation to low salinity conditions (Garcon et al. 2022). Na^**+**^/K^**+**^-ATPase, a transmembrane protein, utilizes ATP energy to drive the active transport of Na^**+**^ and K^**+**^ across the cell membrane at a 3:2 ratio, exchanging intracellular Na^**+**^ for K^**+**^ and thereby maintaining the electrochemical gradient and osmotic balance (Giffard-Mena et al. 2024). Under low salinity stress, the activity of Na^**+**^/K^**+**^-ATPase typically increases to increase the cell capacity to regulate Na^**+**^ and K^**+**^ concentrations, ensuring stability in the internal and external cellular environments (Leone et al. 2012).

Carbonic anhydrase plays a role at another level; it is a rate-limiting enzyme that catalyzes the reversible reaction between carbon dioxide (CO_2_) and water (H_2_O) to form carbonic acid (H_2_CO_3_), which rapidly dissociates into hydrogen ions (H^**+**^) and bicarbonate ions (HCO_3_-)(Mendonca et al. 2007). Under low-salinity conditions, the activity of carbonic anhydrase helps *L. vannamei* adjust their blood and intracellular pH to adapt to the osmotic pressure changes caused by low salinity. Furthermore, the production of HCO_3_^**−**^ is crucial for the function of the Na^**+**^/H^**+**^ exchanger (NHE), which, in low-salinity environments, facilitates the exchange of intracellular H^**+**^ with extracellular Na^**+**^, further promoting Na^**+**^ absorption and pH balance (Saez et al. 2000).

Various genes and proteins in the Na^**+**^/K^**+**^-ATPase and carbonic anhydrase pathways, such as the α- and β-subunit genes of Na^**+**^/K^**+**^-ATPase and various isozymes of carbonic anhydrase, are regulated under low salinity stress, with changes in expression levels directly affecting the functionality of these pathways. For example, the α subunit of Na^**+**^/K^**+**^-ATPase is the catalytic center, whereas the β subunit is involved in protein stability and transport to the cell membrane. Under low salinity stress, the changes in the expression of these subunits ensure that Na^**+**^/K^**+**^-ATPase can respond effectively to environmental changes, increasing *L. vannamei* adaptability (Fabri et al. 2019).

Additionally, in adapting to low-salinity environments, *L. vannamei* also upregulates specific signaling molecules, such as cAMP response element-binding protein (CREB) and c-Jun N-terminal kinase (JNK), to further regulate the activity and expression of the above key proteins. This regulatory network ensures that *L. vannamei* can maintain intracellular and extracellular ion and acid‒base balance through fine regulation of the Na^**+**^/K^**+**^-ATPase and carbonic anhydrase pathways under low-salinity stress, thus ensuring the normal functioning of physiological processes (Leone et al. 2014).

### Autophagy

Under low-salinity stress, autophagy plays a crucial role in important intracellular degradation and recycling pathways, enabling cells to adapt to adverse environments by degrading damaged proteins and organelles to maintain cellular homeostasis and survival. The initiation and execution of autophagy involve a series of molecular mechanisms, including multiple steps and key genes (Gao et al. 2023).

Under low-salinity stress, *L. vannamei* first detects stress signals, which are transmitted to the autophagy core regulatory network via signals such as those from AMP-activated protein kinase (AMPK) or the mTOR signaling pathway (Ou et al. 2022). AMPK, which is activated under energy stress, promotes the initiation of autophagy, whereas the mTOR pathway typically inhibits autophagy under nutrient-rich or other stress conditions. Under low salinity stress, an increase in AMPK activity or a decrease in mTOR activity triggers autophagy (Zhao et al. 2021a).

The initiation of autophagy requires the formation of Atg1, the first step in the autophagy process. The activation of Atg1 depends on the phosphorylation of one of its members, Atg13, by AMPK and the release of mTORC1-mediated inhibition of Atg1. The activation of Atg1 promotes the subsequent formation of autophagosomes. The Atg9 cycle and Atg2-Atg18 facilitate the transport of membrane precursors, providing the material basis for autophagosome formation. Atg9, the only transmembrane Atg protein, is involved in membrane cycling and the expansion of early autophagosomes (Fig. 2C).

During the maturation of autophagosomes, the covalent conjugation of Atg8-PE (phosphatidylethanolamine) is a key step involving the sequential actions of Atg7 and Atg3, which are enzymes akin to E1 and E2 (Burgoyne 2018). Atg8 is initially processed by Atg4 and then assisted by Atg7 (E1-like enzyme) and Atg3 (E2-like enzyme) to covalently bind to PE, anchoring it to the autophagosome membrane (Kaiser et al. 2013). The formation of Atg8-PE not only promotes the expansion and closure of autophagosomes but also participates in the recognition and packaging of substrates (Cao et al. 2016).

Under low salinity stress conditions in *L. vannamei*, the execution of autophagy is completed through the fusion of autophagosomes with lysosomes, where substrates are degraded and recycled. The key molecules involved in this process include Atg14, a Beclin1-associated PI3KC3 complex subunit that promotes autophagosome maturation and autophagosome–endolysosome fusion (Diao et al. 2015). The entire autophagy pathway plays a central role in the *L. vannamei* response to low salinity stress, with a series of Atg proteins finely regulated to ensure cell stability and survival under nutrient deficiency or environmental stress conditions (Fig. 2C).

### ER stress

Under low salinity stress conditions in *L. vannamei*, the endoplasmic reticulum (ER) stress pathway plays a crucial role in the response to and adaptation to environmental pressures through finely tuned molecular mechanisms. Changes in osmotic pressure within *L. vannamei* induced by low salinity are initially detected by sensors on the ER, triggering an ER stress response. The ER stress pathway includes three signaling branches: PERK (PKR-like ER kinase), ATF6 (activating transcription factor 6), and IRE1 (inositol-requiring enzyme 1) (Duan et al. 2024).

First, the PERK branch slows overall protein synthesis under low salinity stress by phosphorylating eIF2α (eukaryotic initiation factor 2α), reducing the burden on the ER. This process simultaneously activates ATF4 (activating transcription factor 4), promoting the expression of stress response proteins such as antioxidants and molecular chaperones, aiding in maintaining the cellular redox balance and protein folding (Chen et al. 2012).

Second, the ATF6 branch is activated under ER stress conditions and translocated to the Golgi apparatus, where it is cleaved to produce an active form of ATF6. This protein enters the nucleus, activating the expression of numerous ER quality control genes, including those encoding molecular chaperones and components of the ER-associated degradation (ERAD) pathway, further enhancing the ability of the cell to handle misfolded proteins (Zhai et al. 2019).

Finally, the IRE1 branch, as one of the oldest ER stress sensors, is activated upon detection of unfolded proteins, initiating the unfolded protein response (UPR). IRE1, with kinase and endoribonuclease activities at its ER-luminal end, specifically cleaves XBP1 mRNA to produce an active form of the XBP1 transcription factor, which promotes the expression of ER molecular chaperones and lipid biosynthesis enzymes, helping maintain ER function and membrane integrity (Fig. 2D) (Ron and Walter 2007; Walter and Ron 2011).

During this process, various stress response genes, such as GRP78/BiP (glucose-regulated protein 78/binding immunoglobulin protein), CHOP (C/EBP homologous protein), and GADD34 (growth arrest and DNA damage-inducible protein 34), play key roles. They are involved not only in the recognition and processing of misfolded proteins but also in regulating cell death programs to adapt to or resist the cellular damage caused by low-salinity stress (Huang et al. 2023).

### Physiological and molecular responses of penaeid shrimp to high salinity stress

In contrast to low-salinity stress environments, high-salinity environments subject penaeid shrimp to hyperosmotic pressure, resulting in cellular dehydration and hemolymph concentration. To counteract these effects, shrimp initiate multiple physiological and molecular responses aimed at preserving osmotic balance. As shown in Fig. 2, active water excretion is reduced, and ion efflux is promoted through transporters such as Na⁺/K⁺-ATPase and Na⁺/K⁺/2Cl⁻ cotransporters. Under high salinity, Na⁺/K⁺-ATPase activity in gill tissues tends to decrease, limiting further ion influx and stabilizing intracellular conditions (Shekhar et al. 2014). The regulation of transmembrane water movement is further supported by changes in aquaporin (AQP) expression, as reported for multiple AQP paralogs in *L. vannamei* under salinity challenges (Wang et al. 2022a).

At the signaling level, activation of the p38 MAPK pathway and upregulation of heat shock proteins (HSPs) help maintain protein stability and cellular homeostasis under osmotic stress (Giffard-Mena et al. 2024). Antioxidant enzyme activities are also modulated under hypersalinity (often showing enzyme- and tissue-dependent patterns), although the response profiles can differ from those under hyposalinity (Luo et al. 2022) .

Consistent with these findings, our recent omics analysis of Penaeus monodon under high-salinity exposure revealed significant transcriptional reprogramming (Huang et al. 2025). Genes associated with ion efflux, including Na⁺/H⁺ exchangers and Cl⁻ channels, were upregulated, whereas aquaporin transcripts were selectively downregulated, reflecting active strategies to limit water loss. In addition, pathways related to glycolysis and fatty acid β-oxidation were activated, indicating an increased energy demand during osmotic adjustment. Metabolomic profiling further revealed remodeling of the membrane lipid composition, suggesting structural adaptations at the cellular level. Together, these adjustments highlight a multifaceted response that enables penaeid shrimp to survive and maintain physiological function in hypersaline environments, including coastal salt ponds and inland hypersaline systems. In addition to osmoregulatory and metabolic adjustments, salinity stress also reshapes immune competence and cellular redox homeostasis; the following section summarizes immune and antioxidant responses that accompany low-salinity acclimation.

## Immune and antioxidant responses to low-salinity stress

Despite fewer studies on hypersalinity than on hyposalinity, the rapid expansion of inland low-salinity farming makes hypo-osmotic acclimation a priority for aquaculture. A sudden decrease in external salinity challenges ion balance and redox homeostasis. Gill-centered osmoregulation relies on coordinated ion-transport systems (e.g., Na⁺/K⁺-ATPase, V-type H⁺-ATPase and acid–base transporters), which are widely implicated in crustacean hyposalinity responses and are frequently highlighted in shrimp transcriptomic/physiological studies (Freire et al. 2008). Hyposalinity is also associated with altered immune performance in shrimp, and oxidative burst/ROS production is a recognized component of crustacean hemocyte immune responses (often involving NADPH oxidase-like complexes) (Wang and Chen 2005). Consistent with this coupling, the Keap1–Nrf2 axis functions as a conserved redox-responsive module and has been characterized in penaeid shrimp, supporting its inclusion as a plausible integrator of antioxidant and immune signaling under salinity stress (Wang et al. 2021). Together, these observations support an integrated view in which osmoregulatory energetics, ROS signaling, and innate immune transcriptional programs are co-regulated during hyposalinity acclimation.

### Immune responses

Under low salinity stress conditions, the differential expression of immune-related proteins in the immune system of spotted *L. vannamei* reflects how salinity stress directly affects the immune function of *L. vannamei*. These proteins include scleroproteins, lysozymes, antilipopolysaccharide factors (ALFs), and others, whose expression adjustments modify *L. vannamei* response strategies to salinity stress and influence their ability to fight pathogens (Gu et al. 2018).

The upregulation of scleroproteins plays a protective role in the immune response of *L. vannamei*, forming a protective barrier by increasing the thickness and hardness of the outer cuticle, reducing the possibility of pathogen invasion and preventing excessive loss of water and ions (Liu et al. 2018). Moreover, the regulation of scleroproteins may involve signaling processes, potentially through the modulation of pathways such as Toll-like receptor (TLR) and NF-κB, thereby impacting the transmission of immune-related signals and strengthening defenses against pathogens (Deepika et al. 2014).

The upregulation of lysozymes reflects the defensive response of *L. vannamei* to salinity stress, increasing their antibacterial ability against bacteria and reducing the risk of pathogen infection. By hydrolyzing critical chemical bonds in bacterial cell walls, lysozymes directly cause bacterial dissolution, constituting an important antibacterial component of the *L. vannamei* immune system (Sangklai et al. 2024). The regulation of antilipopolysaccharide factors (ALFs) under low salinity stress directly enhances the defensive ability of *L. vannamei*. By binding and neutralizing bacterial lipopolysaccharides, ALF prevents bacterial growth and spread, and its upregulation under salinity stress conditions helps to clear pathogens from the environment, maintaining the homeostasis of the immune system (Li et al. 2019). Immune-related proteins play a vital role in *L. vannamei* salinity tolerance, adjusting immune defense strategies, enhancing antimicrobial capabilities, and protecting *L. vannamei* from external environmental and pathogenic threats, thereby promoting survival and adaptation in low-salinity environments (K et al., 2021). Recent transcriptomic data from *Penaeus monodon* indicated that low salinity exposure triggered significant upregulation of Toll-like receptor genes and downstream NF-κB signaling components (Huang et al. 2025). These findings suggest that osmotic stress may prime innate immune pathways even in the absence of pathogens.

Importantly, immune activation under hyposalinity is tightly coupled to redox control. On the one hand, osmotic disturbance and membrane depolarization can elevate ROS (e.g., via NADPH oxidase and mitochondrial sources), which acts as a second messenger to facilitate immune signaling (e.g., TLR/NF-κB) and effector induction. On the other hand, immune effector processes (including prophenoloxidase-related reactions and respiratory-burst-like activities) can further increase ROS load, thereby increasing the requirement for antioxidant buffering. Thus, antioxidant responses should be interpreted as an integral component of low-salinity immune adaptation that preserves hemocyte and gill-cell function and prevents oxidative injury during immune priming.

### Antioxidant defense mechanisms

#### Nrf2-ARE antioxidant response pathway and gene regulation under low-salinity stress

The Nrf2-ARE antioxidant response pathway is a key mechanism by which cells defend against environmental stress, particularly under low-salinity conditions, to maintain the oxidative stress balance. Nrf2 serves as the central transcription factor of this pathway, playing a crucial regulatory role in the expression of a suite of antioxidant and electrolyte balance genes. Nrf2 exerts its regulatory function by binding to the antioxidant response element (ARE) (Chotphruethipong et al. 2023). Beyond detoxifying ROS, Nrf2-regulated antioxidants can modulate inflammatory tone by constraining ROS-dependent amplification of immune signaling (e.g., NF-κB-associated programs), thereby supporting balanced defense under hyposalinity.

Under unstressed conditions, Nrf2 is sequestered in the cytoplasm, bound to Keap1, and kept in an inactive state prone to degradation. However, under oxidative stress induced by low salinity, the conformation of Keap1 changes, leading to the release of Nrf2 from Keap1, thereby stabilizing it. The stabilized Nrf2 subsequently translocates to the nucleus and binds to ARE, a critical step in regulating the expression of antioxidant genes (Wang et al. 2021).

In the nucleus, Nrf2 binding to the ARE triggers the activation of several antioxidant enzyme-encoding genes, including superoxide dismutase (SOD), catalase (CAT), glutathione peroxidase (GPx), and heme oxygenase-1 (HO-1). The proteins produced from these genes increase the ability of cells to resist oxidative stress, helping them cope with potential oxidative damage under low salinity stress.

Furthermore, this pathway has a feedback regulatory mechanism. Newly synthesized antioxidant proteins can inhibit the activity of Nrf_2_ to maintain the balance of the cellular redox state via feedback. This finely tuned regulatory mechanism not only protects cells from oxidative damage caused by low-salinity stress but also prevents the cellular damage that could arise from an overactive response.

#### Antioxidant defense molecular mechanisms

The oxidative stress faced by *L. vannamei* in low-salinity environments not only tests their physiological adaptability but also triggers a series of finely tuned antioxidant defense responses. These responses involve multilevel signal transduction and gene regulatory networks aimed at efficiently clearing excessive reactive oxygen species (ROS) to protect cells from oxidative damage.

Initial Clearance of Superoxide Radicals The activity of superoxide dismutase (SOD) forms the first line of defense against ROS. SOD primarily converts superoxide radicals (O_2_·^−^) into hydrogen peroxide (H_2_O_2_) and oxygen (O_2_), effectively preventing lipid peroxidation and protein oxidative damage caused by superoxide radicals. During this process, *L. vannamei* SOD genes (such as SOD1 and SOD2) are upregulated to meet the increased antioxidant demand (Chen et al. 2023).

The processing of hydrogen peroxide involves the critical roles of catalase (CAT) and glutathione peroxidase (GPx). CAT directly breaks down H_2_O_2_ into water and oxygen, while GPx uses GSH as a reductant to reduce H_2_O_2_ to water, simultaneously producing oxidized glutathione (GSSG). During this stage, the expression of the CAT and GPx genes in *L. vannamei* is upregulated, increasing the ability of the shrimp to handle H_2_O_2_ (Sharif et al. 2008).

Nonenzymatic antioxidants, including vitamins C and E as well as GSH, assist in cellular ROS clearance by directly reacting with free radicals. The antioxidative actions of vitamins C and E are primarily in preventing lipid peroxidation of cell membranes, whereas the reductive role of GSH in the GPx catalytic reaction further strengthens the antioxidant defense network (Yu et al. 2023).

Under low-salinity stress, the activation of the Nrf_2_-ARE signaling pathway crucially regulates the expression of genes encoding antioxidant enzymes and nonenzymatic antioxidants. The nuclear translocation of Nrf_2_ and its binding to ARE not only promote the expression of genes such as SOD, CAT, and GPx but also activate genes involved in GSH synthesis, such as γ-glutamylcysteine synthetase (γ-GCS) and glutathione reductase (GR), enhancing the overall cellular antioxidative capacity. Additionally, other signaling pathways, such as the PI3K/Akt and MAPK pathways, also participate in regulating the expression of these antioxidant genes, providing *L. vannamei* with a comprehensive antioxidant protection network (Fig. 2A).

These immune–redox mechanisms also provide a rationale for targeted nutritional regulation to support energy allocation, membrane stability, and antioxidant capacity during low-salinity acclimation (see Section "Dietary interventions for osmotic stress in low-salinity penaeid shrimp aquaculture").

## Dietary interventions for osmotic stress in low-salinity penaeid shrimp aquaculture

Mechanistically, low-salinity acclimation increases ATP demand for gill ion transport (e.g., Na⁺/K⁺-ATPase/CA; Section "Physiological And Molecular Responses Of Penaeid Shrimp To Low-Salinity Stress") and elevates ROS signaling that requires Nrf2-dependent antioxidant buffering (Section "Immune and antioxidant responses to low-salinity stress"). Therefore, nutritional regulation provides a practical lever to support energy supply, membrane integrity, and immune–redox balance during hyposalinity challenge.

In low-salinity farming, dietary interventions should be interpreted as supportive measures whose effects depend on the specific endpoint measured (growth vs. stress resilience). To avoid conflating general growth nutrition with stress-tolerance evidence, Table 2 summarizes only nutritional interventions with direct experimental support for improved low-salinity stress resilience in penaeid shrimp (e.g., survival, immune/antioxidant indices, or osmoregulatory capacity).

**Table 2:** Nutritional interventions with experimental evidence for improved low-salinity stress resilience in penaeid shrimp

Nutrient	Salinity	Requirement in feed	Findings	References
Cholesterol	25	2–4 g/kg	Optimal cholesterol improves survival, immune response, and antioxidant capacity under low salinity stress	(Cheng et al. 2006)
l-ascorbyl-2-polyphosphate	25	0.1%	Enhances growth and antioxidant status, providing stress resistance in low salinity conditions	(Chen et al. 2017)
Myo-inositol	15	0.02%	Vital for stress tolerance and helps in shell formation in *L. vannamei*	(Chen et al. 2018a)
Mg^2+^	2	2.60–3.46 g/kg	Necessary for optimal growth and osmoregulation in low salinity; interacts with dietary protein levels	(Cheng et al. 2005)
K^+^	4	1.48%	Adequate potassium boosts *L. vannamei* osmoregulatory capacity	(Liu et al. 2013)

Specifically, cholesterol and phospholipid-related inputs can help stabilize membrane structure during osmotic disturbance, vitamin C derivatives (e.g., L-ascorbyl-2-polyphosphate) support antioxidant buffering, and K⁺/Mg^2^⁺ supplementation can enhance ion-balance control and osmoregulatory capacity under hyposalinity. Myo-inositol has also been reported to support salinity stress tolerance. Together, these evidence-supported strategies provide practical options for reducing stress-related losses and improving robustness in low-salinity aquaculture, while broader diet formulation for maximal growth should be guided by separate growth-focused nutrition studies.

## Future research directions and challenges

### Molecular osmosensing

A central priority is to identify and functionally validate upstream osmosensing mechanisms in penaeid shrimp. Key questions include which membrane-associated sensors or receptor systems initiate hyposalinity responses, how Ca^2^⁺ entry/handling routes and lipid-dependent membrane remodeling contribute to early signal encoding, and how these inputs are integrated to control transcriptional programs during acclimation. Progress will require tissue- and cell-type–resolved approaches in osmoregulatory organs (especially gills), coupled with causal perturbation (e.g., RNAi or genome editing where feasible) and quantitative readouts of ion balance, volume regulation, and stress tolerance.

### Omics integration

Beyond single-layer profiling, future work should integrate multi-omics (transcriptomics, proteomics/phosphoproteomics, metabolomics, and lipidomics) with time-series designs to map regulatory flow from perception to effector function. Standardized salinity-challenge regimes and harmonized phenotyping (e.g., hemolymph osmolality, gill transporter activity, immune and redox indices) will improve comparability across studies and species. High-throughput platforms for parallel monitoring of stress-response genes (including shrimp liquid array–type tools) can support rapid candidate prioritization, but their value will be maximized when linked to mechanistic validation and functional outcomes. In crustaceans, the practical utility of array-based screening can be constrained by incomplete gene annotation, probe specificity/cross-hybridization, and limited standardization across tissues and salinity-challenge protocols; therefore, key signals should be confirmed using RNA sequencing (RNA-seq)/quantitative PCR (qPCR) and coupled to functional assays whenever possible.

### Genetic improvement

Genetic improvement should combine quantitative genetics with mechanistic insight to develop salinity-tolerant stocks without compromising growth, reproduction, or disease resistance. Priorities include leveraging inter- and intra-species variability through comparative studies, identifying robust markers and candidate loci (e.g., via association mapping and genomic selection), and evaluating trade-offs under realistic multi-stressor farming conditions. Comparative genomic studies among *L. vannamei*, *P. monodon*, and *Penaeus (Marsupenaeus) japonicus* are now increasingly feasible because chromosome-level genome resources have been generated for these penaeid shrimp (Uengwetwanit et al. 2021; Wei et al. 2025; Zhang et al. 2019), together with earlier draft comparative resources (Yuan et al. 2018). Synteny-aware comparisons, gene-family evolution analyses, and selection scans across species can help prioritize conserved versus lineage-specific mechanisms of salinity tolerance, with emphasis on gill-centered osmoregulatory effectors (e.g., Na⁺/K⁺-ATPase, V-type H⁺-ATPase, NKCC/NHE/anion exchangers, carbonic anhydrase), Ca^2^⁺/lipid signaling modules, and immune/redox regulators. Within species, integrating population genomics across breeding lines/strains with standardized salinity-challenge phenotypes will further enable mapping of genotype-by-environment effects and support marker-assisted/genomic selection for improved salinity tolerance. Where candidate genes are strongly supported, targeted editing strategies may accelerate validation and breeding, but should be coupled to rigorous performance and biosafety assessment across life stages and environments. However, in crustacean research, genome editing is still limited by practical bottlenecks, including challenges in obtaining and manipulating large numbers of synchronized early embryos, variable delivery efficiency (e.g., microinjection/electroporation), mosaicism and low germline transmission, and the current lack of robust pipelines for generating stable edited lines. Accordingly, CRISPR-Cas9 is presently more feasible as a tool for gene-function validation and proof-of-concept testing, while selective breeding/genomic selection and other deployable approaches remain essential for near-term stock improvement.

### Sustainable aquaculture management

Finally, sustainable management should translate mechanistic knowledge into practical mitigation of salinity fluctuations, which are expected to become more frequent and less predictable under climate change (e.g., sea-level intrusion, altered rainfall/runoff patterns, and extreme weather events). This includes stable water-quality control (e.g., salinity buffering and monitoring), adoption of advanced culture systems such as recirculating aquaculture systems (RAS), and management protocols that integrate nutrition, health monitoring, and stress reduction. Aligning feeding strategies (e.g., lipid and micronutrient profiles supporting membrane integrity and redox balance) with osmotic demand, together with early-warning indicators of stress, can reduce mortality and improve productivity under variable salinity regimes.

## Conclusion

This review synthesizes the current knowledge on the physiological and molecular mechanisms facilitating salinity adaptation in penaeid shrimp. The synergistic regulation of ion transport, osmoregulatory processes, antioxidant defenses, and stress signaling pathways, such as the MAPK and PI3K/Akt pathways, forms the foundation of their resilience to environmental fluctuations, particularly in low-salinity conditions. As depicted in Fig. 3, these interconnected responses illustrate an integrated response that maintains cellular homeostasis and physiological function. Despite substantial advancements, the complexity of these regulatory networks suggests that critical aspects of salinity adaptation remain unresolved. Focused research into the molecular architecture of these pathways, especially those pertinent to hypoosmotic stress, will be crucial for enhancing breeding strategies and management practices. A deeper mechanistic understanding will ultimately support climate-resilient penaeid shrimp aquaculture by informing biomarker-based monitoring, targeted breeding, and management strategies that maintain performance under climate-change-driven salinity variability and extremes.

## Data Availability

Not applicable.

## Abbreviations

NF-κB: Nuclear factor kappa-light-chain-enhancer of activated B cells
PI3K: Phosphoinositide 3-kinase
Akt: Protein kinase B
mTOR: Mechanistic target of rapamycin
JNK: C-Jun N-terminal kinase
AP-1: Activator protein 1
Na^+^/K^+^ -ATPase: Sodium/potassium-adenosine triphosphatase
CA: Carbonic anhydrase
cAMP: Cyclic adenosine monophosphate
CREB: CAMP response element-binding protein
ROS: Reactive oxygen species
KEAP1: Kelch-like ECH-associated protein 1
Nrf2: Nuclear factor erythroid 2-related factor 2
SOD: Superoxide dismutase
ALF: Antilipopolysaccharide factor
Toll: Toll-like receptor
TLR: Toll-like receptor
V-type H⁺-ATPase: Vacuolar-type hydrogen-adenosine triphosphatase
AMPK: AMP-activated protein kinase
cGMP: Cyclic guanosine monophosphate
ATP: Adenosine triphosphate
TCA: Tricarboxylic acid cycle
